# Case report: *ex vivo* tumor organoid drug testing identifies therapeutic options for stage IV ovarian carcinoma

**DOI:** 10.3389/fonc.2023.1267650

**Published:** 2024-01-04

**Authors:** Marwah Al-Aloosi, Amanda M. Prechtl, Payel Chatterjee, Brady Bernard, Christopher J. Kemp, Rachele Rosati, Robert L. Diaz, Lauren R. Appleyard, Shalini Pereira, Alex Rajewski, Amber McDonald, Eva J. Gordon, Carla Grandori

**Affiliations:** ^1^ SEngine Precision Medicine, Seattle, WA, United States; ^2^ Earle A. Chiles Research Institute, Providence Cancer Institute, Portland, OR, United States; ^3^ Division of Human Biology, Fred Hutchinson Cancer Center, Seattle, WA, United States; ^4^ Private Health Management, Los Angeles, CA, United States

**Keywords:** low grade serous ovarian cancer, functional precision medicine, tumor organoids, medium-throughput drug screen, fulvestrant, everolimus

## Abstract

Patients presenting with stage 4 ovarian carcinoma, including low-grade serous disease, have a poor prognosis. Although platinum-based therapies can offer some response, these therapies are associated with many side effects, and treatment resistance often develops. Toxic side effects along with disease progression render patients unable to receive additional lines of treatment and limit their options to hospice or palliative care. In this case report, we describe a patient with an unusual case of metastatic low-grade serous ovarian cancer with some features of high-grade disease who had received four previous lines of treatment and was suffering from atelectasis, pulmonary embolism, and hydronephrosis. A CLIA-certified drug sensitivity assay of an organoid culture derived from the patient’s tumor (PARIS^®^ test) identified several therapeutic options, including the combination of fulvestrant with everolimus. On this treatment regimen, the patient experienced 7 months of stable disease and survived nearly 11 months before succumbing to her disease. This case emphasizes the clinical utility of *ex vivo* drug testing as a new functional precision medicine approach to identify, in real-time, personalized treatment options for patients, especially those who are not benefiting from standard of care treatments.

## 1 Introduction

Low-grade serous ovarian carcinoma (LGSOC) comprises less than 5% of ovarian cancers (1). LGSOC usually presents in young women and has unique morphological and molecular features that distinguish it from high-grade tumors (2). Patients who have LGSOC with cancer cells that are limited to the ovary have an excellent prognosis with surgery alone, but most LGSOCs have spread beyond the ovaries and have a poor prognosis (3). Standard of care management for ovarian cancers includes cytoreductive surgery, and for stage 1C and stages 2–4, the addition of platinum-based chemotherapy is indicated (2, 4). However, LGSOC patients generally have poor responses to platinum-based chemotherapies in the neoadjuvant, adjuvant, and relapsed settings, resulting in an unmet need for additional systemic treatment options (5, 6).

Treatments that target hormone receptors are an attractive option, as studies have shown that ~70% of LGSOCs are positive for estrogen receptor (ER) and ~30% are positive for progesterone receptor (PR), defined as weak (1% to 50% of tumor cell nuclei) or strong (≥50%) (7). Hormonal therapy is available for LGSOC as adjuvant, maintenance, and salvage therapy, and data suggest that patients treated with maintenance hormone therapy may have similar outcomes to those treated with maintenance chemotherapy (8). However, despite promising outcomes achieved with these therapies, rates of overall response and progression-free survival (PFS) indicate that they may not work for all patients and may fall short in terms of long-term disease management (9, 10). A variety of additional therapeutic combinations have been proposed to treat LGSOC, including the addition of CDK4/6 inhibitors to hormone therapy regimens like letrozole or fulvestrant, which have improved overall survival rates in patients with metastatic ER-positive breast cancer (11–13).

Patient-derived tumor organoids (PDTOs) have recently been developed to enable *ex vivo* functional testing, including drug screening, of a patient’s tumor cells (14–16). PDTOs retain biologic features and genetic alterations from the originating tumor but also share the entire germline profile as well as any treatment history (17). Because these variables can affect drug sensitivity and response to therapy, controlling for them could enhance the predictive accuracy of patient-derived models relative to other cancer models that are genetically unrelated to any given patient. The PARIS^®^ assay is a CLIA-certified, medium-throughput drug sensitivity assay that employs organoids cultured directly from solid tumors to test drugs or drug combinations in real-time for their potential efficacy (15–19). A report suggesting possible treatment options is then provided to the oncologist in a clinically relevant time frame.

In this case report, we describe a patient with LGSOC whose disease progressed despite surgical intervention and several lines of chemo- and hormonal therapies and who was unable to tolerate further chemotherapy. Tumor organoids were derived from a core biopsy of an abdominal metastatic lesion that was superficial on the right flank and easily accessible and subjected to both single-agent and combination drug sensitivity testing (17, 18). The PARIS^®^ test results identified several additional treatment options including ceritinib, lapatinib, and neratinib, as well as drug combinations, including the ER antagonist, fulvestrant, plus the mTOR inhibitor, everolimus. This combination has shown efficacy in treating hormone therapy-resistant, hormone receptor-positive, EGF-receptor-positive, and HER2-negative breast cancer in postmenopausal patients (20), but to our knowledge, it is not widely used to treat ovarian cancer. Based on the PARIS^®^ test results, the patient was treated with fulvestrant and everolimus and experienced reduced/stabilized CA-125 levels and stable disease for 7 months until she succumbed to her disease after 11 months.

## 2 Case description

### 2.1 Patient history

**Figure 1:**
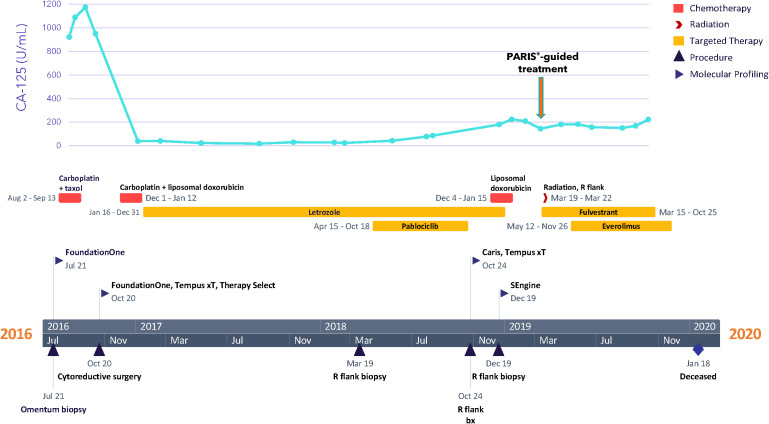
Clinical timeline.

A 27-year-old woman, G1P1A0, presented with bloating and abdominal distension for several weeks, along with oligomenorrhea. Imaging studies showed evidence of clinical-stage IIIC ovarian carcinoma. The patient underwent a CT-guided omental biopsy, and pathology revealed metastatic grade 1 ovarian papillary serous carcinoma with high-grade foci. The patient received three cycles of neoadjuvant chemotherapy with taxol and carboplatin, followed by an exploratory laparotomy, radical resection for tumor debulking, total abdominal hysterectomy, bilateral salpingo-oophorectomy, rectosigmoid resection, partial resection of the transverse colon with re-anastomosis, partial ileal resection with re-anastomosis, and descending colostomy in October of 2016 (**Figure 1**). Her postoperative course was complicated by ileus and by pulmonary embolism, for which the patient received anticoagulation therapy. For adjuvant therapy, the patient switched to carboplatin and liposomal doxorubicin for three cycles and achieved stable disease. In January 2017, the patient started taking the aromatase inhibitor letrozole as maintenance therapy; in March 2018, palbociclib was added to letrozole due to disease progression and the emergence of a right flank mass. This treatment was selected based on the loss of *CDKN2A* noted in genomic profiling of the tumor, discussed below. However, palbociclib was held after two cycles due to grade 3 fatigue. The dose was reduced for the following cycle and terminated after 25 weeks, when the patient was admitted for small bowel obstruction. Six weeks later, the patient started liposomal doxorubicin; however, she received only two cycles due to disease progression that involved recurrent pleural effusion, requiring multiple thoracenteses. Thereafter, the patient suffered from increased flank pain, and imaging studies in February 2019 (about 5 weeks after discontinuing liposomal doxorubicin) showed disease progression and the development of left-sided hydronephrosis.

### 2.2 Tumor stage, pathology, and genomics

**Figure 2:**
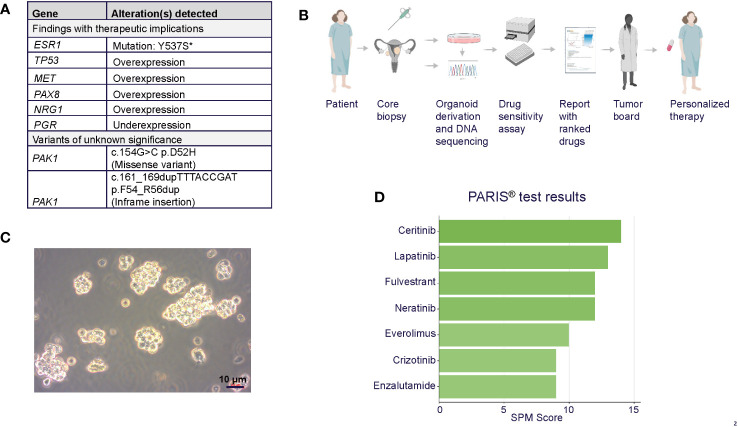
**(A)** Summary of tumor molecular profiling findings with therapeutic implications. *ESR1 mutation was confirmed in organoids. **(B)** PARIS^®^ drug sensitivity assay workflow, including organoid generation from core biopsy, characterization, and report generation. Figure generated using Biorender. **(C)** Brightfield photomicrograph of the patient’s cultured tumor organoids. Scale bar = 10 µm. **(D)** Table of top-scoring drugs in green from the PARIS^®^ assay.

The specific diagnosis for this patient was metastatic papillary serous carcinoma, stage IIIC LGSOC. The tumor exhibited classic low-grade serous morphology with prominent micropapillary features, and nuclear features were >95% low-grade. Foci of more pronounced atypia were noted with some increased mitotic activity, and p53 immunostaining was heterogeneous, consistent with wild-type p53. Additional molecular diagnostics (FoundationOne, December 2016) on a tumor sample from the omentum collected during surgery revealed a *CDKN2A* loss, wild-type TP53, KRAS, NRAS, and BRAF, and a microsatellite stable, mismatch repair proficient, PD-L1-negative tumor with a low mutational burden, indicating that this patient would likely not benefit from immune checkpoint inhibition. No significant germline variants were detected (OvaNext, July 2016), and no somatic mutations in *BRCA1* and *BRCA2* were identified (FoundationOne, December 2016). Further molecular testing (Caris MI Profile) on a right flank tissue sample from October 2018, after 9 months of letrozole, showed that the sample was ER positive, PR negative, and had acquired a somatic pathogenic alteration in the *ESR1* gene (Y537S), suggesting a possible resistance mechanism to letrozole (**Figure 2A**) (21). RNA expression analysis (Tempus xT) on the same tissue further identified overexpression of *TP53*, *MET*, *PAX8*, and *MUC16* (CA125) and underexpression of *PGR*. Full lists of genes included in molecular profiling tests are included in **Supplementary Results**.

### 2.3 Patient-derived tumor organoid-based drug testing

The patient was referred for the PARIS^®^ test after exhausting all other standard of care treatment options. In December 2018, a core biopsy from an abdominal wall metastasis was obtained and shipped to SEngine Precision Medicine (**Figures 2B, C**). The sample was enriched for tumor cells and expanded as a 3D organoid culture for the drug screening assay; detailed methods for organoid culture have previously been described (17, 18). The *ESR1* mutation present in the biopsy tissue was confirmed in the organoids by targeted sequencing (**Supplementary Materials**). The screening assay consisted of a custom drug panel consisting of 12 single agents (cabozantinib, ceritinib, cobimetinib, crizotinib, enzalutamide, everolimus, fulvestrant, lapatinib, neratinib, palbociclib, ribociclib, and sorafenib) and five drug combinations informed by drugs that indicated a response in preliminary testing. Each drug was selected based on the genetic landscape of LGSOC, the genetic profile of this patient’s tumor, and the physician’s request. The drug combination study employed fulvestrant as a sensitizer agent, used at low concentrations, as a measure of the organoids for this patient (IC30). Organoids were then exposed to single drugs at six different concentrations, with or without the addition of fulvestrant. The assay was performed in 384-well plates, and the read-out was Cell Titer Glo measuring ATP concentration in the media as an indicator of cell viability, as previously reported. Drug combination methods were as described (17) and validated in animal PDX models.

The results of the drug screens were read after 6 days of incubation (**Figure 2D**; **Table 1**; **Supplementary Table S2**). The drugs were ranked from the most effective (SPM 15) to the least effective (SPM 1) with a proprietary metric, with scores of 15 to 9 considered active drugs. Exceptional and good single-agent drug responses were observed to ceritinib (SPM 14), lapatinib (SPM 13), fulvestrant (SPM 12), and neratinib (SPM 12), with low responses to everolimus (SPM 10), crizotinib (SPM 9), and enzalutamide (SPM 9). Cobimetinib (SPM 6) indicated a lack of response, while results for sorafenib and palbociclib were not evaluable. Given this patient’s pathogenic mutation in the estrogen receptor gene *ESR1*, which may cause resistance to aromatase inhibitors (22), the selective estrogen receptor degrader (SERD) fulvestrant (Faslodex) was of particular interest and was used as the sensitizing agent for a subsequent five-drug combination screen consisting of fulvestrant plus either neratinib, lapatinib, palbociclib, ribociclib, or everolimus (**Table 2**).

**Table 1:** Single-agent PARIS^®^ test drug screen results.

Drug	Target	*C*_max_	IC_50_	SPM
Ceritinib	ALK, IGF-1R, ROS1	1.43E−06	1.10E−06	14
Lapatinib	EGFR, HER2	4.04E−06	1.30E−06	13
Fulvestrant	Selective estrogen receptor degrader	2.08E−08	NA	12
Neratinib	EGFR, HER1, HER2, HER4	2.14E−07	8.50E−08	12
Everolimus	mTORC1	3.86E−08	7.60E−06	10
Crizotinib	ALK, ROS1, MET	9.48E−07	5.60E−06	9
Enzalutamide	Androgen receptor antagonist	3.57E−05	1.00E−05	9

A list of the drugs that indicated sensitivity according to the PARIS^®^ test was ranked using the SPM score as single drugs. Drug name, gene product target, and maximal serum observed dose (C_max_) as obtained from the literature; all drugs included are FDA-approved. SPM, SEngine Precision Medicine.

**Table 2:** Combination agent PARIS^®^ test results.

Drug	Target	*C*_max_	IC_50_	Single agent AUC	Fulvestrant combination AUC	Absolute difference AUC
Everolimus	mTORC1	3.90E−08	7.60E−06	0.63	0.53	0.11
Lapatinib	EGFR, HER2	4.00E−06	1.30E−06	0.55	0.44	0.10
Ribociclib	CDK4, CDK6	7.10E−06	1.30E−06	0.6	0.51	0.09
Neratinib	EGFR, HER1, HER2, HER4	2.14E−07	8.50E−08	0.43	0.37	0.06

PARIS^®^ testing using a combination of fulvestrant at 1 μM, the pretested IC_30_ concentration for this PDTO, along with either everolimus, lapatinib, ribociclib, or neratinib. The combinations are ranked by the largest differential area under the curve (AUC) obtained using six concentrations of each drug (10 μM, 3.16 μM, 1 μM, 316 nM, 100 nM, and 31.6 nM). Only the drugs that had enhanced activity with fulvestrant are shown.

Despite the low response to everolimus in the single agent screen, this drug was included in the combination testing because it is approved for combination treatment with an ER antagonist for breast cancer and would thus be easier for the patient to obtain. In addition, our prior research found that the combination of fulvestrant plus everolimus was synergistic in a breast cancer patient. Combinations of fulvestrant with neratinib, lapatinib, ribociclib, and everolimus all demonstrated some degree of additive effect, with the best response seen with the HER2 inhibitors lapatinib and neratinib and the mTOR inhibitor everolimus. The combination of fulvestrant and palbociclib did not display an additive response. The evaluation of potential additive or enhanced effects of the drug combination was carried out in consideration of the sensitivity in relation to the overall sensitivity of the combination (single agent AUC) as well as the absolute difference in AUC (Δ AUC) with and without fulvestrant, as shown in **Table 1**. The results indicated that none of the drug combinations were enhanced, but instead, there were additive effects (less than ~10% increased sensitivity when the agents were combined, see ΔAUC column). A CLIA-certified test report describing these results was sent to the treating oncologist 43 days after the sample was received. Additional details about this test can be found in the **Supplementary Materials** and in previous preclinical research papers (15, 16, 23–25).

### 2.4 Post-PARIS^®^ test

Based on genomic profiling and PARIS^®^ test findings, along with consultation with the patient’s oncologist and additional LGSOC experts, treatment with fulvestrant (500 mg on days 1, 15, 29, and subsequently every 28 days) was initiated in March of 2019, followed by palliative radiotherapy for the right flank mass (30 Gy in 10 sessions) the next week and placement of a nephroureteral stent in April 2019. Based on the patient’s tumor organoid drug combination screen, everolimus (10mg, daily) was added to fulvestrant in May 2019. It is noted that the patient received approval from her insurance company for this treatment. However, the malignant pleural effusion resulted in complete right lobe atelectasis, with scans in October showing disease progression. Fulvestrant was discontinued at the end of the month, and everolimus was discontinued a month later, when the patient’s condition deteriorated further. The patient was given antibiotics and hospitalized 1 month later due to severe shortness of breath. Although a decision was made to start the combination of carboplatin, gemcitabine, and bevacizumab, the treatment was not initiated because the patient passed away 1 month later, at 30 years of age. Overall, since the start of fulvestrant and subsequent addition of everolimus 2 months later, the patient’s CA-125 level stabilized (**Figure 1**), and she experienced disease control for 7 months and an overall survival of 11 months.

## 3 Discussion

Ovarian cancers are the second most common cancer of the female reproductive system and are associated with the highest risk of cancer-related death, with most women presenting with advanced-stage disease (26, 27). LGSOC tumors respond poorly to platinum-based chemotherapies (28), making them challenging to treat when there is residual disease following cytoreductive surgery (3, 8, 29). Thus, there is an unmet need to explore targeted treatment options for this subset of patients in the era of personalized medicine.

In this case, a young female patient with LGSOC who had disease progression after surgery and multiple lines of therapy, including neoadjuvant and adjuvant chemotherapies, adjuvant aromatase inhibitors, and CDK4/6 inhibitor treatment, sought further options to help treat her disease. Comprehensive molecular profiling of this patient’s tumor provided information about several other important biomarkers. The patient was not a candidate for immune checkpoint inhibitors (ICI), based on the PD-L1-negative, microsatellite-stable, and mismatch repair-proficient status of the tumor, along with the loss of the cell-cycle regulatory gene *CDKN2A.* This tumor suppressor gene, which is commonly altered in many human cancers, has also been shown to be a marker for poor response to ICI (21). Notably, however, a somatic mutation in the *ESR1* gene was identified, which is significant because breast tumors with *ESR1* mutations have been shown to be resistant to letrozole both alone and in combination with other agents, including the PI3Kα inhibitor alpelisib (21, 30).

Tumor tissue was submitted for PARIS^®^ testing to identify personalized treatment options with the potential to extend the life of this young patient. The results of the PARIS^®^ test on tumor organoids derived from the patient’s metastatic tissue identified multiple candidate single agent and combination treatment options, including fulvestrant plus everolimus. Studies in breast and gynecological cancers have shown promise for each of these agents in ER-positive cancers. For example, *ESR1* mutations do not result in resistance to fulvestrant in patients with metastatic breast cancer (22) as they do with letrozole. In fact, breast tumors harboring *ESR1* mutations have demonstrated greater sensitivity to selective estrogen receptor modulators such as tamoxifen and fulvestrant and to the combination of these endocrine therapies with CDK4/6, PI3K, or mTORC1 inhibitors (31).

It has been established that the PI3K-AKT-mTORC1 pathway plays an important role in endocrine resistance through ligand-independent activation of ER (31) and that one possible adaptive mechanism of resistance to PI3K inhibitors is stimulation of ER activity (32). Therefore, targeting PI3K and mTORC1 by combining their inhibitors with endocrine therapies can be of additive efficacy in endocrine-resistant and *ESR1*-mutated breast cancer (31). Clinical evidence has shown that the combination of fulvestrant and the mTOR inhibitor everolimus extended PFS in patients with breast cancer who became resistant to aromatase inhibitor therapy (20, 33). In the phase II PrE0102 trial, patients treated with everolimus plus fulvestrant had a PFS of 10.3 months, compared with 5.1 months in patients treated with placebo plus fulvestrant. In the phase II MANTA trial, PFS was extended for patients treated with fulvestrant plus everolimus (12.3 months) compared with fulvestrant alone (5.4 months) or fulvestrant plus the mTOR inhibitor vistusertib (7.6 months) (33). The addition of everolimus to letrozole in recurrent gynecologic cancers has also had promising results in heavily pretreated patients with ER-positive cancers (34, 35). It is noteworthy that novel agents are being explored in hormone-resistant breast cancers that harbor *ESR1* mutations, including giredestrant, proxalutamide, and enobosarm (36).

In addition to the combination of fulvestrant with everolimus, the PARIS^®^ test identified several other targeted drugs, including enzalutamide, an oral androgen receptor inhibitor (37), as well as lapatinib and neratinib, which target members of the EGFR family.

Based on the results of the PARIS^®^ test, the patient started fulvestrant in March 2019, and 2 months later, everolimus was added. Her disease remained stable until late October 2019; she ultimately succumbed to her cancer in January 2020. With the treatments identified by the PARIS^®^ test, the patient was able to experience 7 months of stable disease with manageable toxicities. This additional time of stable disease was notable given that the patient harbored many risk factors that are associated with poor prognosis, including being ≤ 35 years of age, having residual disease at the end of primary therapy, and lacking an alteration in the MAPK pathway (38–40).

A limitation of this approach is that challenges are often encountered in obtaining drugs that show effectiveness for individual patients but that are not approved for their specific cancer type. This issue has emerged alongside various precision oncology approaches to cancer treatment and must be urgently addressed by regulatory organizations and payers to enable patients to get the most effective treatments possible.

This case report highlights the successful application of the PARIS^®^ test, a tumor organoid-based drug sensitivity assay, to identify effective targeted therapies for a patient with LGSOC who had progressed on multiple chemo- and targeted therapies. Together with other recent reports showing exceptional responses to organoid-guided therapies in patients who have failed standard of care (15, 19), this demonstrates that *ex vivo* functional testing is a novel precision medicine tool with clinical utility, especially for cancer types that have low responses to standard treatments, such as LGSOC. Given the rarity of this type of disease, this personalized *ex vivo* testing provides an avenue to identify treatments outside of conventional clinical trials. Using organoid-based drug testing to identify targeted therapies could dramatically influence a patient’s outcome and, if employed earlier in the disease course, could preserve the overall patient wellness and quality of life while enhancing their chances for complementary treatment modalities such as immune-oncology interventions toward potential cures (15, 19, 24).

## Data availability statement

The original contributions presented in the study are included in the article/**Supplementary Material**. Further inquiries can be directed to the corresponding authors.

## Ethics statement

The studies involving humans were approved by and managed by Advarra IRB (Pro00036350) under SEngine IRB protocol (SE_IRB_001). The studies were conducted in accordance with the local legislation and institutional requirements. Written informed consent for participation was not required from the participants or the participants' legal guardians/next of kin because SEngine provides a CLIA-certified test to cancer patients following physicians ordering request and also gives the option to patients to sign a consent to be included in an ongoing study enabling collection, aggregation and analysis of molecular and clinical data as they relate to the test. Written informed consent was not obtained from the individual for the publication of any potentially identifiable images or data included in this article because the patient passed away before manuscript writing was initiated. However, this publication does not contain identifiable information related to this patient. SEngine IRB protocol states in the Inclusion/Exclusion criteria the following: “Deceased patients that have passed away after the biological sample has been sent for a PARIS test and before they were able to sign consent, will automatically be included in the research study.

## Author contributions

MA-A: Methodology, Writing – review & editing. AP: Methodology, Writing – review & editing. PC: Formal analysis, Methodology, Writing – review & editing. BB: Methodology, Writing – review & editing, Formal analysis. CK: Writing – original draft. RR: Methodology, Writing – review & editing. RD: Formal analysis, Methodology, Writing – review & editing. LA: Methodology, Writing – review & editing. SP: Formal analysis, Writing – review & editing. AR: Formal analysis, Writing – review and editing. AM: Writing – review & editing, Supervision. EG: Supervision, Writing – original draft. CG: Supervision, Writing – original draft, Conceptualization.
