# Supplementary material for: Case report: ex vivo tumor organoid drug testing identifies therapeutic options for stage IV ovarian carcinoma
Source: Front Oncol. 2024 Jan 4;13:1267650. doi: 10.3389/fonc.2023.1267650 (PMC10794297; doi:10.3389/fonc.2023.1267650)
Supplement: Supplementary file 1 [file DataSheet_1.pdf]

Supplementary Material

Case Report: *Ex vivo* tumor organoid drug testing identifies therapeutic options for stage IV ovarian carcinoma

Marwah Al-Aloosi[1], Amanda M. Prechtl[1], Payel Chatterjee[1], Brady Bernard[1,2], Christopher J. Kemp[3], Rachele Rosati[1], Robert L. Diaz[1], Lauren R. Appleyard[1], Shalini Pereira[1], Alex Rajewski[1], Amber McDonald[4], Eva J. Gordon[4*], Carla Grandori[1*]

[1] SEngine Precision Medicine, Seattle, WA, United States

[2] Earle A. Chiles Research Institute, Providence Cancer Institute, Portland, OR, USA

[3] Division of Human Biology, Fred Hutchinson Cancer Center, Seattle, WA

[4] Private Health Management, Los Angeles, CA, United States

***Correspondence:**

Eva J. Gordon
egordon@privatehealth.com

Carla Grandori
cgrandori@senginemedicine.com

1 Supplementary Methods

1.1 The PARIS® test

Oncologists order the PARIS® test by filling out a requisition form provided by SEngine Precision Medicine. Patients are contacted for an optional consent to the IRB research protocol established at SEngine to enable clinical research, the use of residual material for research, and authorization for medical records to enable clinical research. This patient gave authorization to SEngine Precision Medicine to obtain original medical data.

Organoids are established from surgical excisions, body fluids such as ascites, or biopsies following CLIA certified standard operating procedures. Once an organoid culture reaches a purity level of greater than 70%, they are subjected to drug screening. This patient's culture reached 70% purity after 7 days. A custom panel of 12 drugs was selected for the patient from a library of over 200 oncology agents validated for activity. The SEngine drug library includes FDA-approved and experimental oncology drugs, chemotherapeutics, hormone antagonists, and small-molecule inhibitors. The patient's organoid-based drug sensitivity was measured through a series of standard drug response metrics (IC50 and area under the curve – AUC) as well as proprietary algorithms. Drugs receive an SPM score ranging from 15 to 1, with 15 being the most effective. Drugs that score below 9 are considered not active. A CLIA-certified test report with these results was sent to the oncologist. Additional background can be found in previous pre-clinical research papers (16,23-25).

1.2 Targeted sequencing

The known pathogenic ESR1 mutation that was observed in the tissue biopsy was confirmed in the organoids with targeted sequencing with SNP genotyping (Genewiz). SNP ID rs1057519717 was used to confirm the adjacent mutation of ESR1 Y537S.

2.1 Supplementary results

Supplementary Table 1. Summary of molecular testing performed.

Molecular Test	Date of Specimen Collection	Specimen Site	Results	Genes tested
FoundationOne	7-21-2016	Omentum	*CDKN2A* loss<br>Microsatellite stable<br>MMR proficient<br>Low TMB<br>PD-L1 Negative	DNA sequences for 315 genes and rearrangements in 28 genes<br>(Full list in Suppl. Fig. 1)
FoundationOne	10-20-2016	Omentum	*CDKN2A* loss<br>Microsatellite stable<br>MMR proficient<br>Low TMB	DNA sequences for 315 genes and rearrangements in 28 genes<br>(Full list in Suppl. Fig. 1)
Tempus xT	10-20-2016	Abdominal wall	Overexpression: *TP53*, *MET*, *PAX8*, and *MUC16* (CA125)<br>Underexpression: *PGR*<br>PD-L1 negative<br>Low TMB<br>Microsatellite stable	DNA sequences for 595 genes and full transcriptome RNA sequencing<br>(Full list in Suppl. Fig. 2)
Tempus xT	10-24-2018	Right flank	*ESR1* mutation (Y537S)<br>Overexpression: *TP53*, *MET*, *PAX8*, and *MUC16* (CA125)<br>Underexpression: *PGR*<br>Low TMB<br>Microsatellite stable<br>PD-L1 negative	DNA sequences for 596 genes and full transcriptome RNA sequencing<br>(Full list in Suppl. Fig. 3)
Caris MI Profile	10-24-2018	Right flank	*ESR1* mutation (Y537S)<br>ER-positive<br>PR-negative<br>Low TMB<br>Microsatellite stable<br>MMR proficient<br>PD-L1 negative	DNA sequences for 163 genes and copy number alterations for 39 genes<br>(Full list in Suppl. Fig. 4)

ER, estrogen receptor; MMR, mismatch repair; PR, progesterone receptor; TMB, tumor mutational burden.

Supplementary Figure 1. FoundationOne Gene List

GENES ASSAYED IN FOUNDATIONONE

FoundationOne is designed to include all genes known to be somatically altered in human solid tumors that are validated targets for therapy, either approved or in clinical trials, and/or that are unambiguous drivers of oncogenesis based on current knowledge. The current assay interrogates 315 genes as well as introns of 28 genes involved in rearrangements. The assay will be updated periodically to reflect new knowledge about cancer biology.

DNA Gene List: Entire Coding Sequence for the Detection of Base Substitutions, Insertion/Deletions, and Copy Number Alterations

ABL1	*ABL2*	*ACVR1B*	*AKT1*	*AKT2*	*AKT3*	*ALK*	*AMER1 (FAM123B)*	*APC*	*AR*
ARAF	*ARFRP1*	*ARID1A*	*ARID1B*	*ARID2*	*ASXL1*	*ATM*	*ATR*	*ATRX*	*AURKA*
AURKB	*AXIN1*	*AXL*	*BAP1*	*BARD1*	*BCL2*	*BCL2L1*	*BCL2L2*	*BCL6*	*BCOR*
BCORL1	*BLM*	*BRAF*	*BRCA1*	*BRCA2*	*BRD4*	*BRIP1*	*BTG1*	*BTK*	*C11orf30 (EMSY)*
CARD11	*CBFB*	*CBL*	*CCND1*	*CCND2*	*CCND3*	*CCNE1*	*CD274*	*CD79A*	*CD79B*
CDC73	*CDH1*	*CDK12*	*CDK4*	*CDK6*	*CDK8*	*CDKN1A*	*CDKN1B*	*CDKN2A*	*CDKN2B*
CDKN2C	*CEBPA*	*CHD2*	*CHD4*	*CHEK1*	*CHEK2*	*CIC*	*CREBBP*	*CRKL*	*CRLF2*
CSF1R	*CTCF*	*CTNNA1*	*CTNNB1*	*CUL3*	*CYLD*	*DAXX*	*DDR2*	*DICER1*	*DNMT3A*
DOT1L	*EGFR*	*EP300*	*EPHA3*	*EPHA5*	*EPHA7*	*EPHB1*	*ERBB2*	*ERBB3*	*ERBB4*
ERG	*ERRFI1*	*ESR1*	*EZH2*	*FAM46C*	*FANCA*	*FANCC*	*FANCD2*	*FANCE*	*FANCF*
FANCG	*FANCL*	*FAS*	*FAT1*	*FBXW7*	*FGF10*	*FGF14*	*FGF19*	*FGF23*	*FGF3*
FGF4	*FGF6*	*FGFR1*	*FGFR2*	*FGFR3*	*FGFR4*	*FH*	*FLCN*	*FLT1*	*FLT3*
FLT4	*FOXL2*	*FOXP1*	*FRS2*	*FUBP1*	*GABRA6*	*GATA1*	*GATA2*	*GATA3*	*GATA4*
GATA6	*GID4 (C17orf39)*	*GLI1*	*GNA11*	*GNA13*	*GNAQ*	*GNAS*	*GPR124*	*GRIN2A*	*GRM3*
GSK3B	*H3F3A*	*HGF*	*HNF1A*	*HRAS*	*HSD3B1*	*HSP90AA1*	*IDH1*	*IDH2*	*IGF1R*
IGF2	*IKBKE*	*IKZF1*	*IL7R*	*INHBA*	*INPP4B*	*IRF2*	*IRF4*	*IRS2*	*JAK1*
JAK2	*JAK3*	*JUN*	*KAT6A (MYST3)*	*KDM5A*	*KDM5C*	*KDM6A*	*KDR*	*KEAP1*	*KEL*
KIT	*KLHL6*	*KMT2A (MLL)*	*KMT2C (MLL3)*	*KMT2D (MLL2)*	*KRAS*	*LMO1*	*LRP1B*	*LYN*	*LZTR1*
MAGI2	*MAP2K1*	*MAP2K2*	*MAP2K4*	*MAP3K1*	*MCL1*	*MDM2*	*MDM4*	*MED12*	*MEF2B*
MEN1	*MET*	*MITF*	*MLH1*	*MPL*	*MRE11A*	*MSH2*	*MSH6*	*MTOR*	*MUTYH*
MYC	*MYCL (MYCL1)*	*MYCN*	*MYD88*	*NF1*	*NF2*	*NFE2L2*	*NFKBIA*	*NKX2-1*	*NOTCH1*
NOTCH2	*NOTCH3*	*NPM1*	*NRAS*	*NSD1*	*NTRK1*	*NTRK2*	*NTRK3*	*NUP93*	*PAK3*
PALB2	*PARK2*	*PAX5*	*PBRM1*	*PDCD1LG2*	*PDGFRA*	*PDGFRB*	*PDK1*	*PIK3C2B*	*PIK3CA*
PIK3CB	*PIK3CG*	*PIK3R1*	*PIK3R2*	*PLCG2*	*PMS2*	*POLD1*	*POLE*	*PPP2R1A*	*PRDM1*
PREX2	*PRKAR1A*	*PRKCI*	*PRKDC*	*PRSS8*	*PTCH1*	*PTEN*	*PTPN11*	*QKI*	*RAC1*
RAD50	*RAD51*	*RAF1*	*RANBP2*	*RARA*	*RB1*	*RBM10*	*RET*	*RICTOR*	*RNF43*
ROS1	*RPTOR*	*RUNX1*	*RUNX1T1*	*SDHA*	*SDHB*	*SDHC*	*SDHD*	*SETD2*	*SF3B1*
SLIT2	*SMAD2*	*SMAD3*	*SMAD4*	*SMARCA4*	*SMARCB1*	*SMO*	*SNCAIP*	*SOCS1*	*SOX10*
SOX2	*SOX9*	*SPEN*	*SPOP*	*SPTA1*	*SRC*	*STAG2*	*STAT3*	*STAT4*	*STK11*
SUFU	*SYK*	*TAF1*	*TBX3*	*TERC*	*TERT (promoter only)*	*TET2*	*TGFBR2*	*TNFAIP3*	*TNFRSF14*
TOP1	*TOP2A*	*TP53*	*TSC1*	*TSC2*	*TSHR*	*U2AF1*	*VEGFA*	*VHL*	*WISP3*
WT1	*XPO1*	*ZBTB2*	*ZNF217*	*ZNF703*					

DNA Gene List: For the Detection of Select Rearrangements

ALK	*BCL2*	*BCR*	*BRAF*	*BRCA1*	*BRCA2*	*BRD4*	*EGFR*	*ETV1*	*ETV4*
ETV5	*ETV6*	*FGFR1*	*FGFR2*	*FGFR3*	*KIT*	*MSH2*	*MYB*	*MYC*	*NOTCH2*
NTRK1	*NTRK2*	*PDGFRA*	*RAF1*	*RARA*	*RET*	*ROS1*	*TMPRSS2*		

Additional Assays: For the Detection of Select Cancer Biomarkers

Microsatellite status

Tumor Mutation Burden

Supplementary Figure 2. Tempus xT Gene List from October 2016 Test.

Heme Related Genes

ARHGAP26	BIRC3	CIITA	DDX3X	ETV6	HDAC1	LEF1	MAPK1	NUP98	POT1	SMARCA1	STAT5B	TCL1A	WHSC1
BCL10	CBLB	CKS1B	DNM2	FBXO11	HDAC4	MAF	MIB1	P2RY8	RAD21	SMC1A	STAT6	TNFRSF17	ZRSR2
BCL11B	CBLC	CSF3R	EBF1	FHIT	HIST1H1E	MAFB	MKI67	PCBP1	RHOA	SMC3	SUZ12	TP63	
BCL7A	CD22	CUX1	ECT2L	FOXO1	HIST1H3B	MALT1	NCOR2	PHF6	SETBP1	SRSF2	TBL1XR1	TRAF3	
BCR	CD70	CXCR4	EPOR	FOXO3	KMT2B	MAP3K7	NT5C2	PIM1	SGK1	STAT5A	TCF3	TUSC3	

Both Heme and Solid Tumor Related Genes

ABCB1	AURKB	CARD11	CDKN2B	EGFR	FANCD2	FLT1	IDH1	KEAP1	MITF	NOTCH1	PIK3R2	RUNX1	STAT3
ABCC3	AXIN1	CBFB	CDKN2C	EP300	FANCE	FLT3	IDH2	KIT	MLH1	NOTCH2	PLCG2	SDHA	STAT4
ABL1	AXL	CBL	CEBPA	EPHA7	FANCF	FLT4	IKBKE	KLHL6	MPL	NPM1	PPP2R1A	SDHB	STK11
AKT1	B2M	CCND1	CHD2	EPHB1	FANCG	FOXL2	IKZF1	KMT2A	MRE11A	NRAS	PRDM1	SDHC	SUFU
AKT2	BAP1	CCND2	CHEK1	ERBB2	FANCL	FOXP1	IL7R	KMT2C	MSH2	NTRK1	PRKAR1A	SDHD	TAF1
AKT3	BARD1	CCND3	CHEK2	ERBB3	FAS	FRS2	INPP4B	KRAS	MSH3	NTRK2	PTCH1	SETD2	TET2
ALK	BCL2	CCNE1	CIC	ERBB4	FBXW7	GATA1	IRF1	LRP1B	MSH6	NTRK3	PTEN	SF3B1	TGFBR2
AMER1	BCL6	CD274	CREBBP	ERG	FGF10	GATA2	IRF4	MAP2K1	MTOR	PALB2	PTPN11	SMAD2	TMPRSS2
APC	BCOR	CD79A	CRKL	ESR1	FGF14	GATA3	IRS2	MAP2K2	MUTYH	PAX5	RAD50	SMAD4	TNFAIP3
AR	BCORL1	CD79B	CRLF2	ETS1	FGF23	GNA11	JAK1	MAP2K4	MYC	PBRM1	RAD51	SMARCA4	TNFRSF14
ARAF	BLM	CDC73	CSF1R	ETV1	FGF3	GNA13	JAK2	MAP3K1	MYCL	PDCD1	RAF1	SMARCB1	TOP1
ARID1A	BRAF	CDH1	CTCF	ETV4	FGF4	GNAQ	JAK3	MCL1	MYCN	PDCD1LG2	RARA	SMO	TP53
ARID2	BRCA1	CDK12	CTNNA1	ETV5	FGF6	GNAS	JUN	MDM2	MYD88	PDGFRA	RB1	SOCS1	TSC1
ASXL1	BRCA2	CDK4	CTNNB1	EWSR1	FGFR1	GRIN2A	KAT6A	MDM4	NF1	PDGFRB	RET	SOX10	TSC2
ATM	BRD4	CDK6	DAXX	EZH2	FGFR2	HGF	KDM5A	MED12	NF2	PDK1	RICTOR	SOX2	TSHR
ATR	BRIP1	CDK8	DDR2	FAM46C	FGFR3	HNF1A	KDM5C	MEF2B	NFE2L2	PIK3CA	RNF43	SPEN	U2AF1
ATRX	BTK	CDKN1B	DNMT3A	FANCA	FGFR4	HRAS	KDM6A	MEN1	NFKBIA	PIK3CG	ROS1	SPOP	VHL
AURKA	CALR	CDKN2A	DOT1L	FANCC	FLCN	HSP90AA1	KDR	MET	NKX2-1	PIK3R1	RPTOR	SRC	WT1
												STAG2	XPO1

Solid Tumor Related Genes

ABL2	C11orf65	CYP3A5	ERCC5	FNTB	HIF1A	HLA-E	IRF2	MTHFR	PDPK1	PTCH2	RSF1	TAP1	WRN
ACTA2	C3orf70	DDB2	ERCC6	FOXA1	HIST1H4E	HLA-F	ITPKB	MTRR	PHOX2B	PTPN13	RUNX1T1	TAP2	XPA
ACVR1B	C8orf34	DICER1	ERRFI1	FOXQ1	HLA-A	HLA-G	KEL	MYB	PIAS4	PTPN22	RXRA	TBC1D12	XPC
AJUBA	CASP8	DIRC2	ETS2	FUBP1	HLA-B	HNF1B	KIF1B	MYH11	PIK3C2B	PTPRD	SCG5	TBX3	XRCC1
APOB	CASR	DIS3	FAM175A	G6PD	HLA-C	HOXB13	KLLN	NBN	PIK3CB	QKI	SDHAF2	TCF7L2	XRCC2
ARHGAP35	CBR3	DIS3L2	FANCB	GALNT12	HLA-DMA	HSPH1	KMT2D	NCOR1	PIK3CD	RAC1	SEC23B	TIGIT	XRCC3
ARID1B	CCDC6	DKC1	FANCI	GATA4	HLA-DMB	IDO1	LAG3	NHP2	PML	RAD51B	SEMA3C	TMEM127	YEATS4
ARID5B	CD19	DPYD	FANCM	GATA6	HLA-DOA	IFIT1	LDLR	NOP10	PMS1	RAD51C	SH2B3	TMEM173	ZFHX3
ASNS	CD40	DYNC2H1	FAT1	GEN1	HLA-DOB	IFIT2	LMNA	NOTCH3	PMS2	RAD51D	SLC26A3	TNF	ZNF217
ATIC	CDKN1A	EGF	FCGR2A	GLI1	HLA-DPA1	IFIT3	LMO1	NQO1	POLD1	RAD54L	SLC47A2	TNFRSF9	ZNF471
ATP7B	CDKN1C	EGLN1	FCGR3A	GPC3	HLA-DPB1	IFNAR1	LYN	NRG1	POLE	RANBP2	SLIT2	TOP2A	ZNF620
AXIN2	CEP57	ELF3	FDPS	GPS2	HLA-DPB2	IFNAR2	LZTR1	NSD1	POLH	RASA1	SLX4	TPM1	ZNF750
BCL2L1	CFTR	ENG	FGF1	GREM1	HLA-DQA1	IFNGR1	MAD2L2	NTHL1	POU2F2	RBM10	SMAD3	TPMT	ZNRF3
BCL2L11	CHD4	EPCAM	FGF2	GRM3	HLA-DQA2	IFNGR2	MAX	NUDT15	PPP1R15A	RECQL4	SMARCE1	TYMS	
BCLAF1	CTC1	EPHA2	FGF5	GSTP1	HLA-DQB1	IFNL3	MC1R	PAK1	PPP2R2A	RINT1	SOD2	UBE2T	
BMPR1A	CTLA4	EPHB2	FGF7	H19	HLA-DQB2	IL10RA	MGMT	PALLD	PPP6C	RIT1	SOX9	UGT1A1	
BTG1	CTRC	ERCC1	FGF8	H3F3A	HLA-DRA	IL15	MLH3	PARK2	PRCC	RNF139	SPINK1	UGT1A9	
BUB1B	CYLD	ERCC2	FGF9	HAS3	HLA-DRB1	IL2RA	MLLT3	PAX3	PREX2	RPL5	SPRED1	UMPS	
C10orf54	CYP1B1	ERCC3	FH	HAVCR2	HLA-DRB5	IL6R	MS4A1	PAX7	PRSS1	RPS15	SYK	VEGFA	
C11orf30	CYP2D6	ERCC4	FLG	HDAC2	HLA-DRB6	ING1	MTAP	PAX8	PRSS2	RPS6KB1	TANC1	WEE1	

Gene Rearrangements by DNA Sequencing

ABL1	BRAF	EWSR1	MYB	NTRK1	PDGFRA	RET
ALK	EGFR	FGFR2	MYC	NTRK3	PML	ROS1
BCR	ETV6	FGFR3	NRG1	PAX8	RARA	TMPRSS2

*MSI is reported for tumor/normal matched samples only.

In addition to reporting on somatic variants, when a normal sample is provided, Tempus reports germline incidental findings on a limited set of variants within genes selected based on recommendations from the American College of Medical Genetics (ACMG) and published literature on inherited cancer syndromes. Patients always have the option to opt out of receiving this information.

Supplementary Figure 3. Tempus xT Gene List from October 2018 Test.

Heme Related Genes

ARHGAP26	BIRC3	CIITA	DDX3X	ETV6	HDAC1	LEF1	MAPK1	NUP98	POT1	SMARCA1	STAT5B	TCL1A	WHSC1
BCL10	CBLB	CKS1B	DNM2	FBXO11	HDAC4	MAF	MIB1	P2RY8	RAD21	SMC1A	STAT6	TNFRSF17	ZRSR2
BCL11B	CBLC	CSF3R	EBF1	FHIT	HIST1H1E	MAFB	MKI67	PCBP1	RHOA	SMC3	SUZ12	TP63	
BCL7A	CD22	CUX1	ECT2L	FOXO1	HIST1H3B	MALT1	NCOR2	PHF6	SETBP1	SRSF2	TBL1XR1	TRAF3	
BCR	CD70	CXCR4	EPOR	FOXO3	KMT2B	MAP3K7	NT5C2	PIM1	SGK1	STAT5A	TCF3	TUSC3	

Both Heme and Solid Tumor Related Genes

ABCB1	AURKB	CARD11	CDKN2B	EGFR	FANCD2	FLT1	IDH1	KEAP1	MITF	NOTCH1	PIK3R2	SDHA	STAT4
ABCC3	AXIN1	CBFB	CDKN2C	EP300	FANCE	FLT3	IDH2	KIT	MLH1	NOTCH2	PLCG2	SDHB	STK11
ABL1	AXL	CBL	CEBPA	EPHA7	FANCF	FLT4	IKBKE	KLHL6	MPL	NPM1	PPP2R1A	SDHC	SUFU
AKT1	B2M	CCND1	CHD2	EPHB1	FANCG	FOXL2	IKZF1	KMT2A	MRE11A	NRAS	PRDM1	SDHD	TAF1
AKT2	BAP1	CCND2	CHEK1	ERBB2	FANCL	FOXP1	IL7R	KMT2C	MSH2	NTRK1	PRKAR1A	SETD2	TET2
AKT3	BARD1	CCND3	CHEK2	ERBB3	FAS	FRS2	INPP4B	KRAS	MSH3	NTRK2	PTCH1	SF3B1	TERT*
ALK	BCL2	CCNE1	CIC	ERBB4	FBXW7	GATA1	IRF1	LRP1B	MSH6	NTRK3	PTEN	SMAD2	TGFBR2
AMER1	BCL6	CD274	CREBBP	ERG	FGF10	GATA2	IRF4	MAP2K1	MTOR	PALB2	PTPN11	SMAD4	TMPRSS2
APC	BCOR	CD79A	CRKL	ESR1	FGF14	GATA3	IRS2	MAP2K2	MUTYH	PAX5	RAD50	SMARCA4	TNFAIP3
AR	BCORL1	CD79B	CRLF2	ETS1	FGF23	GNA11	JAK1	MAP2K4	MYC	PBRM1	RAD51	SMARCB1	TNFRSF14
ARAF	BLM	CDC73	CSF1R	ETV1	FGF3	GNA13	JAK2	MAP3K1	MYCL	PDCD1	RAF1	SMO	TOP1
ARID1A	BRAF	CDH1	CTCF	ETV4	FGF4	GNAQ	JAK3	MCL1	MYCN	PDCD1LG2	RARA	SOCS1	TP53
ARID2	BRCA1	CDK12	CTNNA1	ETV5	FGF6	GNAS	JUN	MDM2	MYD88	PDGFRA	RB1	SOX10	TSC1
ASXL1	BRCA2	CDK4	CTNNB1	EWSR1	FGFR1	GRIN2A	KAT6A	MDM4	NF1	PDGFRB	RET	SOX2	TSC2
ATM	BRD4	CDK6	DAXX	EZH2	FGFR2	HGF	KDM5A	MED12	NF2	PDK1	RICTOR	SPEN	TSHR
ATR	BRIP1	CDK8	DDR2	FAM46C	FGFR3	HNF1A	KDM5C	MEF2B	NFE2L2	PIK3CA	RNF43	SPOP	U2AF1
ATRX	BTK	CDKN1B	DNMT3A	FANCA	FGFR4	HRAS	KDM6A	MEN1	NFKBIA	PIK3CG	ROS1	SRC	VHL
AURKA	CALR	CDKN2A	DOT1L	FANCC	FLCN	HSP90AA1	KDR	MET	NKX2-1	PIK3R1	RPTOR	STAG2	WT1
											RUNX1	STAT3	XPO1

Solid Tumor Related Genes

ABL2	C11orf30	CYP2D6	ERCC4	FNTB	HIF1A	HLA-E	IRF2	MTHFR	PDPK1	PTCH2	RSF1	TAP1	WEE1
ACTA2	C11orf65	CYP3A5	ERCC5	FOXA1	HIST1H4E	HLA-F	ITPKB	MTRR	PHOX2B	PTPN13	RUNX1T1	TAP2	WRN
ACVR1B	C3orf70	DDB2	ERCC6	FOXQ1	HLA-A	HLA-G	KEL	MYB	PIAS4	PTPN22	RXRA	TBC1D12	XPA
AJUBA	C8orf34	DICER1	ERRFI1	FUBP1	HLA-B	HNF1B	KIF1B	MYH11	PIK3C2B	PTPRD	SCG5	TBX3	XPC
APLNR	CASP8	DIRC2	ETS2	G6PD	HLA-C	HOXB13	KLLN	NBN	PIK3CB	QKI	SDHAF2	TCEB1	XRCC1
APOB	CASR	DIS3	FAM175A	GALNT12	HLA-DMA	HSPH1	KMT2D	NCOR1	PIK3CD	RAC1	SEC23B	TCF7L2	XRCC2
ARHGAP35	CBR3	DIS3L2	FANCB	GATA4	HLA-DMB	IDO1	LAG3	NHP2	PML	RAD51B	SEMA3C	TIGIT	XRCC3
ARID1B	CCDC6	DKC1	FANCI	GATA6	HLA-DOA	IFIT1	LDLR	NOP10	PMS1	RAD51C	SH2B3	TMEM127	YEATS4
ARID5B	CD19	DPYD	FANCM	GEN1	HLA-DOB	IFIT2	LMNA	NOTCH3	PMS2	RAD51D	SLC26A3	TMEM173	ZFHX3
ASNS	CD40	DYNC2H1	FAT1	GLI1	HLA-DPA1	IFIT3	LMO1	NQO1	POLD1	RAD54L	SLC47A2	TNF	ZNF217
ATIC	CDKN1A	EGF	FCGR2A	GPC3	HLA-DPB1	IFNAR1	LYN	NRG1	POLE	RANBP2	SLIT2	TNFRSF9	ZNF471
ATP7B	CDKN1C	EGLN1	FCGR3A	GPS2	HLA-DPB2	IFNAR2	LZTR1	NSD1	POLH	RASA1	SLX4	TOP2A	ZNF620
AXIN2	CEP57	ELF3	FDPS	GREM1	HLA-DQA1	IFNGR1	MAD2L2	NTHL1	POU2F2	RBM10	SMAD3	TPM1	ZNF750
BCL2L1	CFTR	ENG	FGF1	GRM3	HLA-DQA2	IFNGR2	MAX	NUDT15	PPARG	RECQL4	SMARCE1	TPMT	ZNRF3
BCL2L11	CHD4	EPCAM	FGF2	GSTP1	HLA-DQB1	IFNL3	MC1R	PAK1	PPP1R15A	RINT1	SOD2	TYMS	
BCLAF1	CTC1	EPHA2	FGF5	H19	HLA-DQB2	IL10RA	MGMT	PALLD	PPP2R2A	RIT1	SOX9	UBE2T	
BMPR1A	CTLA4	EPHB2	FGF7	H3F3A	HLA-DRA	IL15	MLH3	PARK2	PPP6C	RNF139	SPINK1	UGT1A1	
BTG1	CTRC	ERCC1	FGF8	HAS3	HLA-DRB1	IL2RA	MLLT3	PAX3	PRCC	RPL5	SPRED1	UGT1A9	
BUB1B	CYLD	ERCC2	FGF9	HAVCR2	HLA-DRB5	IL6R	MS4A1	PAX7	PREX2	RPS15	SYK	UMPS	
C10orf54	CYP1B1	ERCC3	FH	HDAC2	HLA-DRB6	ING1	MTAP	PAX8	PRSS1	RPS6KB1	TANC1	VEGFA	

Gene Rearrangements by DNA Sequencing

ABL1	BRAF	EWSR1	MYB	NTRK1	PDGFRA	RET
ALK	EGFR	FGFR2	MYC	NTRK3	PML	ROS1
BCR	ETV6	FGFR3	NRG1	PAX8	RARA	TMPRSS2

* Includes promoter region.

In addition to reporting on somatic variants, when a normal sample is provided, Tempus reports germline incidental findings on a limited set of variants within genes selected based on recommendations from the American College of Medical Genetics (ACMG) and published literature on inherited cancer syndromes. Patients always have the option to opt out of receiving this information.

Supplementary Figure 4. Caris MI Profile gene list.

GENES TESTED WITHOUT POINT MUTATIONS OR INDELS BY NGS

ABL1	AKT1	ALK	AMER1	APC	AR	ARAF	ARID2	ATM	ATR	BAP1	BARD1
BCOR	BLM	BMPR1A	BRAF	BRCA1	BRCA2	BRIP1	CARD11	CCND1	CCND2	CCND3	CD79B
CDC73	CDH1	CDK12	CDK4	CDK6	CDKN1B	CDKN2A	CHEK1	CHEK2	CIC	CREBBP	CSF1R
CTNNB1	CYLD	DDR2	DICER1	DNMT3A	EGFR	EP300	ERBB2 (Her2/Neu)	ERBB3	ERBB4	ERCC2	EZH2
FANCA	FANCC	FANCD2	FANCE	FANCF	FANCG	FANCL	FBXW7	FGFR1	FGFR2	FGFR3	FGFR4
FH	FLCN	FLT1	FLT3	FOXL2	FUBP1	GATA3	GNA11	GNA13	GNAQ	GNAS	H3F3A
H3F3B	HIST1H3B	HNF1A	HRAS	IDH1	IDH2	IRF4	JAK1	JAK2	JAK3	KDM5C	KDM6A
KDR (VEGFR2)	KIT	KMT2A	KMT2C	KMT2D	KRAS	LCK	MAP2K1 (MEK1)	MAP2K2 (MEK2)	MAX	MEN1	MET
MITF	MLH1	MPL	MRE11	MSH2	MSH6	MTOR	MUTYH	MYCN	MYD88	NBN	NF1
NF2	NPM1	NRAS	NSD1	NTRK1	NTRK2	NTRK3	PALB2	PBRM1	PDGFRA	PDGFRB	PHOX2B
PIK3CA	PIK3R1	PIM1	PMS1	PMS2	POLE	POT1	PPARG	PPP2R1A	PRDM1	PRKAR1A	PRKDC
PTEN	PTPN11	RAF1	RB1	RET	RNF43	ROS1	SDHAF2	SDHB	SDHC	SDHD	SETD2
SF3B1	SMAD2	SMAD4	SMARCA4	SMARCB1	SMARCE1	SMO	SPOP	SRC	STK11	SUFU	TERT
TP53	TSC1	TSC2	U2AF1	VHL	WRN	WT1					

GENES TESTED WITHOUT COPY NUMBER ALTERATIONS (AMPLIFICATIONS) BY NGS

AKT2	ALK	ARID1A	AURKB	CCND1	CCND3	CCNE1	CD274 (PD-L1)	CDK4	CDK6	CDK8	CDKN2A
CREBBP	CRKL	EGFR	EP300	ERBB2 (Her2/Neu)	EZH2	FGF10	FGF3	FGF4	FGFR1	FGFR2	FGFR3
GATA3	KDR (VEGFR2)	MAP2K1 (MEK1)	MCL1	MDM2	MET	MYC	NF2	NFKBIA	NTRK1	RB1	RICTOR
ROS1	TOP1	WT1									

Supplementary Table 2. All drug results from the PARIS® test.

*Sorafenib was not reported because the curve had poor GOF. GOF, goodness of fit; SPM, SEngine Precision Medicine score.

Drug	Target	Cmax	Inhibition at Cmax	IC50	GOF	AUC	SPM
Ceritinib	ALK, IGF-1R, ROS1	0.00000143	91	1.E-06	0.939834	0.463708	14
Lapatinib	HER2	0.00000404	73	1.E-06	0.978372	0.546801	13
Neratinib	EGFR, HER1, HER2, HER4	2.14E-07	53	9.E-08	0.392985	0.432102	12
Fulvestrant	selective estrogen receptor degrader	2.08E-08	NA	Not Reached	0.459806	0.766484	12
Sorafenib	VEGFR, PDGFR, RAF	0.0000211	NA	Not Reached	0.396155	0.876004	11*
Everolimus	mTORC1	3.86E-08	35	8.E-06	0.728675	0.632184	10
Crizotinib	ALK, ROS1, MET	9.48E-07	18	6.E-06	0.907276	0.741848	9
Enzalutamide	androgen	0.0000357	NA	Not Reached	0.995386	0.985087	9
Cobimetinib	MEK	5.14E-07	52	4.E-07	0.862446	0.562029	6
Palbociclib HCl	CDK4, CDK6	3.08E-07	9	9.E-06	0.813818	0.890205	5
